# Unfolding and modeling the recovery process after COVID lockdowns

**DOI:** 10.1038/s41598-023-30100-5

**Published:** 2023-03-13

**Authors:** Xuan Yang, Yang Yang, Chenhao Tan, Yinghe Lin, Zhengzhe Fu, Fei Wu, Yueting Zhuang

**Affiliations:** 1 Zhejiang University, Hangzhou, China; 2 University of Chicago, Chicago, USA; 3 Zhejiang Huayun Info-Tech Co., Ltd., Hangzhou, China

**Keywords:** Applied mathematics, Computational science, Computer science, Scientific data

## Abstract

Lockdown is a common policy used to deter the spread of COVID-19. However, the question of how our society comes back to life after a lockdown remains an open one. Understanding how cities bounce back from lockdown is critical for promoting the global economy and preparing for future pandemics. Here, we propose a novel computational method based on electricity data to study the recovery process, and conduct a case study on the city of Hangzhou. With the designed *Recovery Index*, we find a variety of recovery patterns in main sectors. One of the main reasons for this difference is policy; therefore, we aim to answer the question of how policies can best facilitate the recovery of society. We first analyze how policy affects sectors and employ a *change-point detection algorithm* to provide a non-subjective approach to policy assessment. Furthermore, we design a model that can predict future recovery, allowing policies to be adjusted accordingly in advance. Specifically, we develop a deep neural network, *TPG*, to model recovery trends, which utilizes the graph structure learning to perceive influences between sectors. Simulation experiments using our model offer insights for policy-making: the government should prioritize supporting sectors that have greater influence on others and are influential on the whole economy.

## Introduction

After the global outbreak of COVID-19, lockdown became a policy commonly used to deter its spread^1–3^. However, it has been unclear how our society would return to life after the lockdown, if it could at all. The lockdown policies have caused the closure of workplaces and educational institutions, leading to a reduced workforce across all economic sectors^4–6^. Many works have studied the socio-economic impacts of the lockdown policies^7–10^, but few study the recovery after lockdown. Moreover, these existing studies focus primarily on how the economy as a whole or a specific sector has been influenced by and recovered from COVID-19^11–15^. There is a general lack of research that studies the recovery patterns of different sectors from the COVID-19 depression. Furthermore, novel computational methods are needed for socio-economic recovery evaluation. In addition, governments often enact a series of policies to promote recovery^16,17^ after the lockdown, yet the specific effects of these policies remain unknown.

To examine in detail how social life recovers after the lockdown is lifted, we propose to investigate the impacts on society from a micro point of view by examining the main sectors that underpin society. Many existing studies use traditional macroeconomic indicators (e.g., GDP) to analyze the socio-economic impact of COVID-19^18–20^. However, these indicators might not be sufficiently accurate and flexible to capture the detailed recovery patterns of different sectors in a specific area. Therefore, we need more granular data sources to understand the recovery process in a fine-grained manner. Since electricity plays an indispensable role in almost all socio-economic activities within cities, electricity consumption data can effectively reflect the socio-economic condition^21–24^. Inspired by this, in this paper, we utilize accurate electricity consumption data per day and per unit to explore the recovery process after lockdown. Our method can be applied to most cities that have experienced lockdowns. Specifically, we take Hangzhou, the fourth-largest metropolitan area in China, as an example to conduct the first large-scale quantitative exploration of the socio-economic recovery after the lifting of a lockdown policy in response to COVID-19.

Our data includes 76 million electricity consumption records from 11,464 organizations (i.e., dedicated electricity users), provided by the State Grid Corporation of China. As shown in Supplementary Table 1, we divide all organizations into 17 sector types (e.g., manufacturing, education, catering, etc.), covering the main sectors of our society. Based on the electricity data, we design a *recovery index* to serve as our basic tool for observing the recovery patterns of different sectors. We find that different recovery patterns occur after a lockdown. In particular, we notice that while lockdown policies constituted a blow to most sectors, they have also brought new opportunities to some sectors (e.g., online sectors and the logistics sector). Although some studies have examined the recovery of different sectors, these works tend to focus on sector in particular, or lack a valid metric that can be used to consistently and precisely assess the recovery of all types of sectors^11,12,25,26^.

As the most important factor impacting sector recovery, government policies directly affect recovery patterns^27–29^. Ineffective policies can slow down recovery and even give rise to substantial costs. To inform the development of future policy, we incorporate the electricity data with the collected policies issued by the governments (Supplementary Table 2) and propose a computational assessment method. Specifically, we adopt a change point detection algorithm, which enables us to automatically identify the sudden changes in the recovery trends of sectors. By comparing the detected change points with the implementation date of policies, we could examine the effect of policies in a non-subjective way. For instance, we determine that the policy of allowing students to return to school on a batch-by-batch basis firmly controlled the recovery of the education sector, allowing it to return steadily to its pre-epidemic state; however, the policy of offering restaurant spending coupons provided only a short-lived boost to the catering sector, which remained depressed afterwards.

Furthermore, we study an important question in policy development: how should we assign policy support to each sector? We aim to address this question by analyzing the socio-economic impact of different support policies. We design a novel deep learning model and and use it to conduct several simulation experiments. Unlike existing recovery predict models, our model utilizes a graph structure learning mechanism, which can capture the correlation between sectors and help to predict recovery trends more accurately. In our simulation experiments, our concern is how much of a boost different sectors will get *if they are given a recovery-promotion policy*. Moreover, we observe how the chain effect works in the society’s recovery^30^. For example, in Hangzhou, the recovery of the catering sector and entertainment sector is largely affected by the recovery of the tourism sector. The government should take these relationships into account when formulating policies.

**Figure 1:**
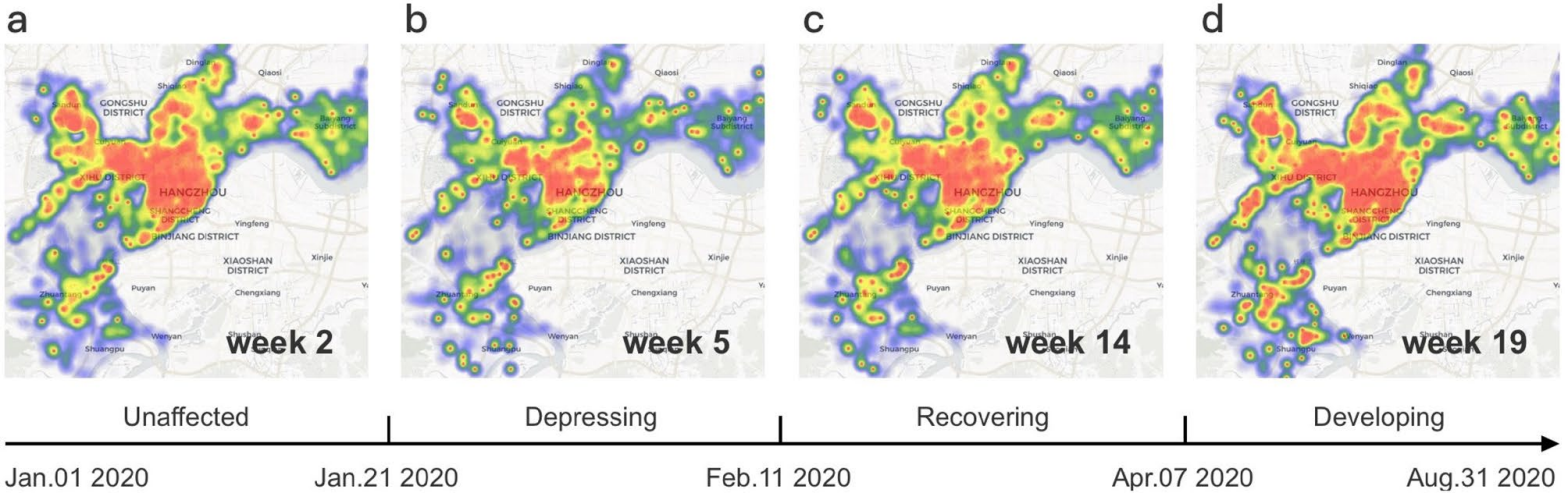
Heat maps illustrating the influence of COVID-19 lockdown on the Hangzhou urban area^31^. Each point in a heat map represents the weekly electricity consumption of an organization. Warmer colors represent greater electricity consumption. (**a**) The heat map of week 2 2020, 1 week before the outbreak of the COVID-19 epidemic (Jan. 8–Jan. 14). (**b**) The heat map of week 5 2020, one week after the outbreak (26 Jan.–4 Feb.). (**c**) The heat map of week 14 2020, ten weeks after the outbreak (Apr 01–Apr. 07). (**d**) The heat map of week 19 2020, 4 months after the outbreak (May 27–Jun. 03).

### Diverse sector recovery patterns

In order to scrutinize the depression and subsequent recovery process of a city after the lockdown policies, we utilize our daily electricity consumption data to design a *Recovery Index*. Compared with existing methods based on commonly used economic data (e.g., GDP data and stock price data) used to observe socio-economic conditions, using electricity data to build our *Recovery Index* offers three main benefits. First, electricity data offers more precision over time. GDP is typically calculated on an annual or quarterly basis^32^, which is much more coarse-grained compared with electricity data that is measured on a daily basis. Most existing works^33–36^ use annual GDP data to analyze the economic impact of COVID-19. However, the socio-economic conditions of the society changed dramatically under the influence of COVID-19 lockdowns, which cannot be reflected by GDP in time. The day-to-day electricity data, enable us to better understand the detailed recovery process after lockdowns and respond to it. Second, electricity data offers more flexibility when evaluating the recovery process of a specific area. Though some indices, such as the Baltic Exchange Dry Index (BDI)^37^ and the Morgan Stanley Capital International (MSCI) World Index (MSCI)^38^ do exist to evaluate the economic condition, they are mainly based on country level. As electricity data is easier to be acquired and calculated on any level of a geographic hierarchy, our designed recovery index can be more flexible in use. Finally, the electricity data offers a unified way to evaluate most sectors of our society. We acknowledge that some existing works use stock price data to evaluate economic impact^39,40^. As most companies are still small local businesses, they do not have publicly traded stock, and thus their conditions cannot be reflected by stock price data. Moreover, some sectors (e.g., education sector and management sector) may not have publicly traded stock either. Since electricity plays an indispensable role almost in all socio-economic activities in cities, its consumption data could offer us a chance to evaluate the recovery patterns of most sectors of our society in a unified way^21–24^.

The recovery index provides a uniform and effective measure of the degree of recovery in different sectors, and serves as a basic tool for our analysis throughout the paper. The recovery index is calculated from the year-on-year change in weekly electricity consumption and adjusted by a range of factors, including weather, development level, and lunar festivals, with a regression model (see details in method).

We apply the recovery index to the analysis of Hangzhou’s recovery. We mainly focuses on the period of greatest socio-economic change before and after the COVID-19 epidemic (Jan. 01, 2020–Aug. 31, 2020). We divide this period according to the recovery index (socio-economic conditions) in Hangzhou into three stages: depressing, recovering, and developing, which are respectively illustrated in Fig. 1. Due to lockdown policies such as strict restrictions on outings and resumption of work, the average recovery index of all organizations continued to fall to 0.589 in the early days of the outbreak, representing a 41.1% decline in the overall economy compared to pre-epidemic levels. We call this stage *Depressing* (Jan. 22, 2020–Feb. 11, 2020). With the COVID-19 epidemic under control, the government gradually lifted lockdown policies, and Hangzhou moved to the *Recovering Stage* (Feb. 12, 2020–Apr. 07, 2020). Sectors began to recover, and by the end of this stage, the average recovery index of organizations reached 1.00. Subsequently, after three months of the outbreak, Hangzhou entered a stable *Developing Stage* (Apr. 08, 2020–Aug. 31, 2020), and most sectors recovered or were in even better condition.

**Figure 2:**
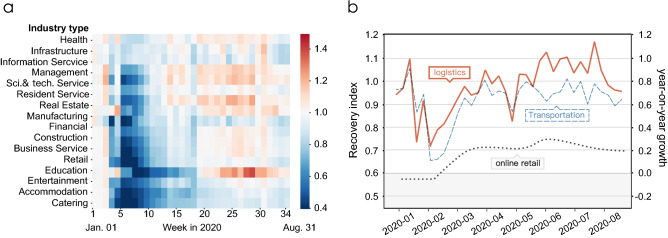
Illustration of recovery patterns of different sectors. (**a**) The recovery index of 17 sectors from January 1, 2020 to August 31, 2020. We ranked these sectors in order of how quickly their recovery index reached one for the first time after week 5. (**b**) Through our case study on the transportation sector, we find that lockdown policies brought new opportunities to the logistics and online sectors. The orange and blue lines show the change in the weekly recovery index of the logistics and transportation sectors respectively (the logistics sector is a sub-sector of the transportation sector). They share the axis on the left of the figure. The grey line shows the monthly year-on-year growth of residents’ online consumption in Hangzhou (right axis). Note that the points of January and February on the grey line indicate the same value, which is the average year-on-year growth of January and February (see details in method).

To further observe the recovery process of different sectors, we group organizations into 17 sectors (Supplementary Table 2) and calculate the average weekly recovery index for organizations in each sector from January to August, respectively. As shown by the recovery index values in Fig. 2, different sectors exhibit different recovery patterns. Among the 17 sectors, health, infrastructure, information service, and management were the sectors least impacted by the lockdown; these organizations are key to protecting people’s livelihoods and managing the epidemic, so they remained functional under the lockdown policies. In comparison, other sectors were hit hard, suffering a decline up to and exceeding 40% under the lockdown. The sectors of scientific and technical service, transportation, resident service, and real estate recovered first from the severe depression, followed by manufacturing, financial, and construction. And the recovery of business service and retail was somewhat slower, while the sectors of education, entertainment, accommodation, and catering exhibited the slowest recovery speed.

From these recovery patterns, we can observe that although the epidemic led to strict mobility restrictions, the transportation sector underwent a paradoxically rapid recovery^41^. According to our observations, the reason lies in one of its sub-sectors: logistics. More specifically, we find that after a short period of depression (31% drop in recovery index in the sixth week), its recovery index quickly reached 0.97 in the eleventh week (Mar. 11, 2020–Mar. 17, 2020), and in most cases, stayed above 1 after the fourteenth week (Apr. 01, 2020–Apr. 07, 2020). In addition to the additional logistics burden caused by the epidemic prevention and control, the more important reason for the increase in logistics is the increase in online shopping. The lockdown and social distance restrictions disrupted consumer habits^42^, with more people becoming inclined to shop online to prevent the exposure risk of offline shopping^43,44^. Consistent with our observations regarding logistics, according to data from the Department of Commerce of Zhejiang Province, Hangzhou’s online retail sector has grown rapidly since March.

### Policy influence on sector recovery patterns

As one of the major cities in China that has been greatly affected by the epidemic, Hangzhou adopted various recovery-promoting policies to actively respond to the COVID-19 epidemic, helping sectors to recover while ensuring that the epidemic is fully under control.

**Figure 3:**
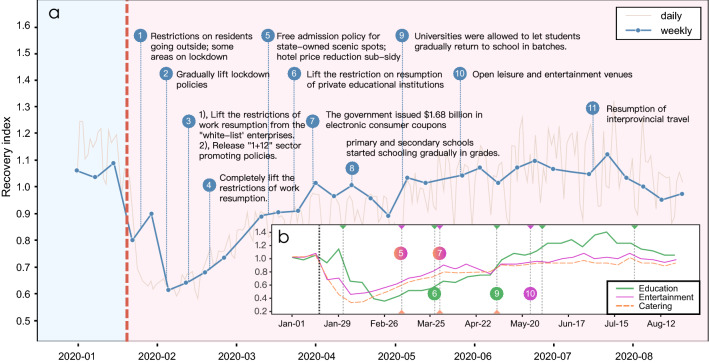
Study of the policies during and after the COVID-19 lockdown in 2020. (**a**) We plot the time points of policy issuance on the curve of the weekly recovery index to observe the impact of the policies. Most of the policies were issued by the Hangzhou or Zhejiang government from Jan. 21 to Aug. 31 2020, and are closely related to the 17 main sectors. The main content of each policy is displayed next to its time point. The red vertical line represents the outbreak. (**b**) We adopt the change-point detection algorithm to identify the sudden changes in each sector’s recovery trend. Change points in the recovery index of education, entertainment, and catering sectors are detected to observe the effects of the policies on each sector. Each vertical dotted line represents a detected change point. The color indicates the sector to which the change point belongs. Each number on a vertical line shows that this change point corresponds to the policy with this number in (**a**).

Examining the specific effect of each policy on different sectors will aid the government in developing better policies. Many existing works have evaluated the effectiveness of lockdown policies with reference to confirmed cases data or mobility data^30,45,46^. When comes to evaluate the effect of recovery (supporting) policies, one common approach is to use questionnaires^15,28^, which is time-consuming and costly. Here, we introduce a timely and non-subjective method involving the change point algorithm to help assess policy effects. Our designed change point algorithm can detect sudden changes in sector recovery trends^47,48^. If the timing of the policy and a detected change-point align (the recovery trend changes because of the external influence of the policy), we can generally assume that the policy has an effect, and further observe how it has made a specific change in the recovery of the sector.

Using this approach, we first examine the education sector (the detected change point results of all sectors are shown in Supplementary Fig. 5). A strict policy by the Hangzhou government was adopted in the education sector. Unlike the early restriction-lifting policy for most enterprises, the Hangzhou government postponed the resumption of educational institutions to prevent transmission of COVID-19 caused by in-person schooling. Thus, although most sectors began to recover after the sixth week, the education sector remained depressed. As shown by the change point detection results in Fig. 3b, the education sector exhibits a trend of steady recovery with policy control, with its change points 2 and 3 corresponding to policies 6 and 9 in Fig. 3a.

In addition to the education sector, the catering and entertainment sectors also fell into a period of significant depression under the lockdown policies. Despite policies being put in place that were designed to stimulate their recovery, the suppressive effect of the lockdown on these two sectors remained long-lasting. The change points 2 and 3 in catering and entertainment correspond to policies 5 and 7 in Fig. 3a. It is worth noting that, after the coupon policy that was implemented on 27 March 2020, these two sectors recovered significantly in a short term (within one week), but exhibited a less promising recovery trend in the following month (the recovery index for the entertainment sector even decreased by 0.16. This suggests that such policies may have only a short-term effect.

### How should governments make policies to promote recovery?

**Figure 4:**
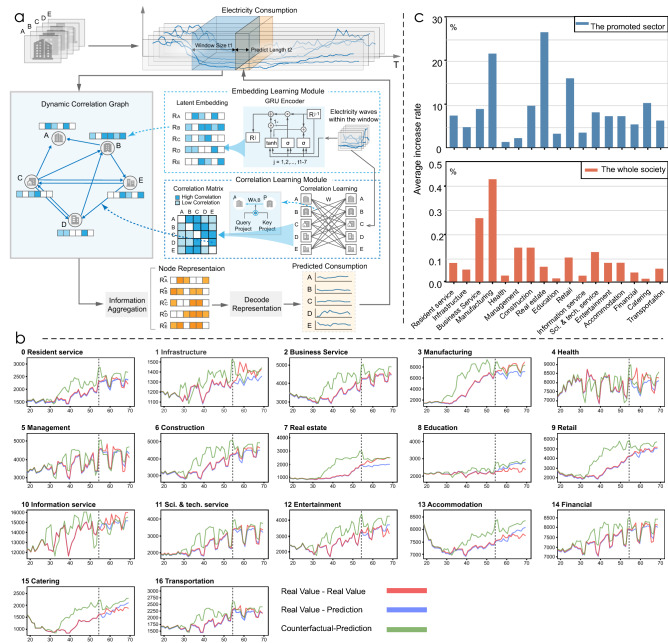
We adopt a graph learning-based time series model to capture the recovery trends of 17 sectors and conduct simulation experiments to explore the effects of support policies. (**a**) Model structure. We first build the correlation graph by simultaneously generating the graph node (organization) features (modeling the temporal information from organizations’ electricity consumption sequences) and build the directed edges (based on the correlations between organizations calculated by the correlation module). We then conduct information aggregation on the learned graph and decode the aggregated representations to obtain predictions. (**b**) The simulation values and model prediction results. The vertical dotted line divides the time series sequence into 56 days and 14 days (we omit dates 0–17 for clarity). The red line is the original electricity consumption series. The blue line is the original consumption series for dates 0–55 and the model prediction for dates 56–69. The green line on the left represents the simulation values of the electricity consumption series for 17 sectors, which simulates the influence of adding promotion policies to each sector from Feb. 05, 2020 (Date 27–Date 55). The right part of the figure is the model predictions with the inputs of simulation values. (**c**) The impact on future recovery if we provide support to different sectors in the simulation experiments. The upper figure is the relative growth rate of electricity consumption for the next 14 days in a sector following the provision of policy support to that sector. The lower figure represents the average relative growth rate of electricity consumption for the next 14 days in all sectors except the policy-promoted sector.

When formulating the policies, to help the overall economy and social life to recover quickly from the epidemic, the following two questions should be carefully considered by public officials: (1) support for which sector will bring the greatest benefit to its recovery? (2) support for which sector will cause the greatest benefit to the recovery of all sectors? To answer these two questions, we aim to model the recovery process of sectors throughout society. Using the daily electricity consumption records of each organization, we propose a time series forecasting model based on dynamic graph learning. Rather than using traditional prediction methods, we design a graph learning module, which is designed to capture the correlations between sectors; accordingly, it allows us to better model sector recovery and reveal the link effects. The proposed model, named TPG, enables us to evaluate the effect of promotion policies on different sectors, which can be very helpful for policy-making.

Specifically, as shown in Fig. 4a, our model first generates a dynamic correlation graph, in which a node represents an organization. We adopt a gate recurrent unit (GRU)^49^ to model the temporal information from past daily electricity consumption series as node features of the graph. Meanwhile, the correlation learning module calculates the correlation weights , which form the basis for building the directed and weighted edges of the graph. Then, on the built dynamic graph, each organization will aggregate information from other organizations and generate its final representation^50^, which will later be decoded into predicted future recovery trends. We calibrate our model by minimizing the Mean Square Error (MSE) between our decoded result and the real daily electricity consumption sequence for the next 14 days.

**Figure 5:**
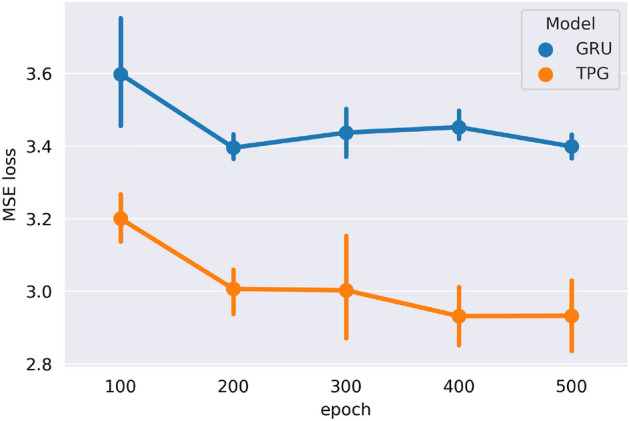
The test results of TPG and the GRU seq2seq model (MSE loss). To evaluate the model performance on the prediction task, we separately train the TPG and the GRU for 100, 200, 300, 400, and 500 epochs on the train set and then test on the test set. Each experiment is repeated at least 5 times. The results illustrate that our model can better capture the recovery trend than traditional model and the graph learning module does capture the valid relationships between sectors.

Our model could precisely predict the future recovery trend (electricity consumption) of almost every sector during the epidemic (from 26 Feb. to 31 Aug. 2020), despite changing policies, recurrent outbreaks of COVID-19, weather, and the interference of holidays. With the help of the correlations we learned, we obtain much better results than when only using the GRU model (Fig. 5). This proves that our model captures the effective correlations between organizations to help model the recovery process. Using the learned model, we can estimate whether we should increase policy support for a sector to accelerate its recovery, along with the impact the policy will have on its future recovery. Furthermore, with the aid of the correlation mechanism we have designed, we can further observe how the recovery of one sector will affect other sectors (i.e., the overall economy).

For each sector, we assume that the government had given greater support to this sector when the economy had just begun to recover from the blow of the lockdown (05 Feb. 2020). As shown in Fig. 4b, we constructed simulation electricity consumption series for each sector by doubling the speed of its recovery from 05 Feb. 2020 (see details in method). Then, in each of our experiments, we change the time series inputs (electricity consumption series) of all organizations belonging to a particular sector to our simulated values, while leaving the inputs of other sectors’ organizations unchanged, and observe how this affects the recovery trend of all sectors over the next fourteen days.

The results of our experiments shown in the upper figure of Fig. 4c answer our first question (”adding support for which sector will yield the greatest benefit to its recovery?”). First, by adding policy support, we observe that each sector will experience a positive impact on future recovery trends. Among them, the manufacturing, real estate, and retail sectors benefit the most from the increased policy support, followed by the catering, construction, and business service sectors. Sectors that were less impacted during the epidemic, such as the health and managements sectors, benefit less from the new increased policy support.

However, when we consider how to promote the recovery of the whole economy and social life rather than a single sector, things change. In the lower part of Fig. 4c, the values represent the impact of adding support for a sector on the overall recovery of all sectors (without the sector itself). In general, we would intuitively assume that those sectors that are most affected by increased policy support will also have the greatest impact on the overall recovery of society. However, our experimental results reject this conjecture. For example, although the catering and business service sectors show a similar boost to their own recovery when being respectively supported by promotion policies (10.8% increase for catering; 9.3% for business service), their contributions to the overall recovery of sectors are quite different. Increased policy support for the business service sector is 27 times more likely to contribute to the overall economy recovery than support for the catering sector. The business service sector includes tourism, management service, and so on, meaning that its recovery is more likely to have a strong chain effect on the recovery of other sectors than the catering sector. For example, as a tourist city, the recovery of Hangzhou’s business service sector is very important for the catering, entertainment, and accommodation sectors. Moreover, although the increased support for the management sector (state administrative agencies, international organizations, etc.) will only increase the recovery of the sector itself by about 2%, the chain effect leads to a significant increase in overall economic recovery (ranked third among all sectors). These results show that the ability of different sectors to drive the recovery of other sectors varies greatly. When the government formulates policies, it should take into account influence relationships, as this will enable it to develop more effective policies to promote the overall recovery of sectors.

## Discussion

Our study introduces large-scale electricity data to investigate how our society might return to life before the lockdown, which allows us to observe the recovery process in a more precise way. In our observations, we design a *recovery index* as the basic tool used to study the recovery patterns of different sectors. The influence of lockdown on different sectors exhibits diverse patterns. For example, due to the change of lifestyle brought about by the lockdown, COVID-19 in fact created new opportunities for the online and logistics sectors. Furthermore, we comb through the timeline of sector-related policies after the implementation of lockdown and study their impacts. We propose to utilize the change point algorithm to verify the influence of various policies on different sectors, which can provide governments with non-subjective, real-time feedback to support policy development. In addition, in order to study which sectors the government should provide policy support to, we construct a graph-learning-based time series model that can precisely predict the future recovery trends of sectors. With this model, we conduct simulation experiments on each sector; the results tell us which sectors are more likely to gain more from supportive policies. Furthermore, we consider correlations between sectors to determine which sectors should be targeted with support policies in order to provide the greatest benefit to overall recovery of our society.

Our datasets have some limitations. First, we do not analyze all sectors because of the insufficient sample sizes of some sectors (see more details in method). Moreover, the time points of the COVID-19 epidemic outbreak and that of the spring festival holiday in China (Jan. 25, 2020) overlap. Although we adjust our observation data to account for the factor of lunar festivals, this interference cannot be completely eliminated. However, our study covers most sectors, meaning that it can generally reflect the recovery of social life and the economy. Intuitively, when considering how to optimize policy-making in terms of socio-economic impact, the government should pay more attention to those sectors that are more likely to be greatly depressed by lockdown, such as manufacturing, entertainment, and catering. However, the government should not blindly formulate support policies based on the extent to which the sector has been hit; instead, it should carefully consider the recovery characteristics of the sector and the inter-dependence between sectors. For instance, the manufacturing sector responds positively to financial support policies, while the catering and entertainment are not greatly assisted by them. The reason for this is that the nature of the catering sector makes it impossible to recover quickly in the short term (people are less willing to go out for meals because of the risk of COVID-19 infection). Rather than offering policies that directly stimulate the catering sector (such as issuing coupons), the government should give priority to supporting the manufacturing sector, etc., to recreate a stable flow of people and thus encourage a return of restaurant customers. Moreover, the chain effect between sectors should be considered in policy-making. Taking Hangzhou as an example, only when the tourism sector (part of the business service sector) is revitalized can the catering and entertainment sectors return to pre-lockdown levels. To maximize the socio-economic recovery of our society, it is recommended that governments give priority to promoting the sectors that have strong chain effects on other sectors.

Our research framework and the proposed model can be applied to other cities. We hope that our research will help cities that are experiencing a lockdown recover more quickly afterwards, and provide guidance for dealing with similar incidents that may occur in the future.

## Method

In the method, we first describe the data that we used in this paper and then introduce the recovery index, change point algorithm, and our graph-learning-based time series model in turn.

### Data

#### Electricity data

The electricity data is offered by the State Grid Corporation of China, the major power supply company in China. Our data includes 11,464 dedicated electricity users and their 76 million daily electricity consumption records. These users are located in the urban areas of Hangzhou. And each dedicated electricity user corresponds to a company or an organization registered in State Grid (anonymous). We mainly analyse the electricity data for eight months around the outbreak of the COVID-19 epidemic (Jan. 01, 2020–Aug. 31, 2020). And we also use the data from Jan. 01, 2019 to Dec. 31, 2019 to supplement our analysis.

The State Grid Corporation of China also provides us with subcategory information for each dedicated electricity user. According to ”Industrial classification for national economic activities”^51^ and subcategory information, we group all organizations into 19 sectors. As the numbers of organizations belonging to the agriculture sector and the mining sector are less than 100, to ensure the representativeness of our sector analysis, the data used in this paper removes the organizations belonging to these two sectors. The detailed classification result is shown in Supplementary Table 1.

#### Policy data

We collect the COVID-19 epidemic-related lockdown policies and 17 sectors related policies during the period from Jan. 21, 2020 to Aug. 31, 2020. The policies are mainly collected from the official portal of Hangzhou Municipal Government^52^ and Zhejiang Government’s Official Web Portal^53^. All collected policies are summarized in Supplementary Table 2, including their timing, content, and government departments that issue these policies.

#### Online retail data

We obtain the monthly year-on-year growth in online consumption data of residents in Hangzhou from the Department of Commerce of Zhejiang Province^54^. In Fig. 2b, the year-on-year growth rates of January and February have the same value, which is the average monthly growth rate of the two months. It is because the Zhejiang Department of Commerce do not have the separate monthly data for January and February.

#### Temperature data

We obtain the daily temperature data of the Hangzhou urban area from the China Meteorological Network. The period of daily temperature data we used ranged from Jan. 01, 2019 to Aug. 31, 2020.

### Recovery index

In this paper, we use *Recovery index* as a uniform metric to evaluate the recovery of each sector. The recovery index is calculated by the electricity data and temperature data with the python language.

### Unadjusted recovery index

The recovery index is a year-on-year change value in weekly electricity consumption, adjusted by temperature, lunar holiday factor, and development level. The unadjusted recovery index is calculated by:

1 $$\begin{aligned} R_{i,w} = E_{i,w}^{2020}/E_{i,w}^{2019} \end{aligned}$$

where $R_{i,w}$ denotes the recovery index for sector *i* at the *w*th week 2020. $E_{i,w}^{2020}$ denotes the weekly electricity consumption value of sector *i* at the *w*th week 2020.

### Adjusted for temperature

The impact of temperature on electricity consumption data is relatively significant. For example, as shown in Supplementary Fig. 1, due to the abnormally high temperature in mid-June, almost all the unadjusted recovery index of sectors sudden rebound. Thus, we first want to remove the interference of temperature in our observation of the recovery degree of sectors. To eliminate such interference on the recovery index, we use a regression model to capture the nonlinear relationship between electricity consumption and temperature,

2 $$\begin{aligned} P_{e_d} = f(T_d) = x_1*T_d+x_2*T_d^2+x_3*T_d^3+b \end{aligned}$$

where *P* represents the predicted value, $e_d$ represents the daily electricity consumption of date *d*, $T_d$ denotes the temperature value of date *d*, $x_1$,$x_2$,$x_3$,*b* represent learnable parameters. The regression model is trained on temperature and electricity data from January to December in 2019, calibrated by minimizing the Mean Square Error (MSE) between the predicted value *P* and the real daily electricity consumption value *E*. Then we adjust the recovery index by the learned function:

3 $$\begin{aligned} e_d^*= & {} e_d / P_{e_d} \end{aligned}$$

4 $$\begin{aligned} R_{i,w}^*= & {} E_{i,w}^{2020*} / E_{i,w}^{2019*} \end{aligned}$$

Here $E_{i,w}^{2020*}$ presents the *i* sector’s electricity consumption in *w*th week 2020 after temperature adjusted. The result is shown in Supplementary Fig. 2, the abnormal rebound recovery in mid-June is corrected.

### Adjusted for lunar festivals

We further consider the interference of lunar festivals. For instance, as the most important and longest holiday in China, the Spring Festival largely affects the consumption of electricity. As it is a lunar festival, it corresponds to different Gregorian dates in 2019 and 2020. As shown in Supplementary Fig. 3, there is an abnormal rise in the first week of February, which is corresponding to the Spring Festival of 2019 (5 Feb. 2019). To eliminate such interference, we match the four weeks around Spring Festival of 2020 (week 2–5) with the four weeks around Spring Festival of 2019 (week 3–6):

5 $$\begin{aligned} R_{i,3-6}^* = E_{i,3-6}^{2020*}/E_{i,4-7}^{2019*} \end{aligned}$$

Besides Spring Festival, we also consider and align Dragon Boat Festival and Qing Ming Festival. After the adjustments, the final recovery index result is shown in Supplementary Fig. 4, which can basically eliminate the interference of lunar festivals. However, as the Spring Festival affects different sectors in different time spans, such interference cannot be completely eliminated.

### Adjusted for development level

We then eliminate the impact of different development levels of different sectors from 2019 to 2020 for the recovery observation. For example, in Supplementary Fig. 2, after a year of economic development, the electricity consumption of the real estate sector at the beginning of 2020 is higher than that of last year. As our aim is only to observe the degree of depression and subsequent recovery of the sector due to the lockdown policy, we adjust the development factors by dividing the electricity consumption data of each sector in 2020 by its development index *D*:

6 $$\begin{aligned} R_i^* = R_i^*/D_i \end{aligned}$$

The development index is evaluated by dividing the total electricity consumption of the first three weeks, which is free from the impact of the epidemic, in 2020 by the total of the same period in 2019:

7 $$\begin{aligned} D_i = E_{i,1-3}^{2020*}/E_{i,1-3}^{2019*} \end{aligned}$$

The adjusted result is shown in Supplementary Fig. 3, from which we can clearly observe the recovery patterns of different sectors are on the same scale.

### Change point algorithm

We adopt a change point algorithm to automatically detect the sudden changes in the recovery index. If a policy is effective for a sector, then the recovery trend of this sector should change after the implementation because of the external influence of the policy. Thus, we validate the effectiveness of one policy by matching the policy timing with the detected change points. We conduct change point detection on our recovery index from Jan. 01, 2020 to Aug. 31, 2020. It is widely considered that change point detection (CPD) is the problem of finding abrupt change points in data when some properties of the time series change^55^. We explore what properties of the time series can be useful in detecting change points. For example, some naive properties like mean, slope, and variance may count. As more general methods, some early works on change point detection^56,57^ locate the shift in the mean of independent and identically distributed (iid) Gaussian variables. Following this intuition, kernel-based change point detection is used in our work to effectively detect the change points in the recovery trend.

Let’s consider $c(\cdot )$ as a cost function to measure the confidence of a piece of time series (recovery trend) that we can regard as heterogeneous. For a piece of time series from change point index on $t_{k}$ to $t_{k+1}$, it can be represented as $x_{t_{k}\ldots t_{k+1}}$. Then the criterion function $C(\cdot )$ is the sum of cost function of the segments sliced from complete time series^58^.

8 $$\begin{aligned} C(\Gamma ,x):=\sum ^{K}_{k=0}c(x_{t_{k}\ldots t_{k+1}}) \end{aligned}$$

where $\Gamma$ is the set of change point index $\Gamma =\{t_1,\ldots ,t_k\}$. And the best change point detection result can be obtained by get the minimization of the criterion $C(\Gamma ,x)$.

### Kernel based change point detection

We adopt kernel-based method^59^ to perform change point detection, as it is a robust non-parametric method when that does not require much prior knowledge. More specifically, we use a Gaussian kernel-based change point detection named Rbf kernel (Radial basis function kernel).

The standard kernel-based change point detection cost function can be given by

9 $$\begin{aligned} c_{kernel}(x_{a\ldots b}):=\sum ^b_{t=a+1}k(x_t,x_t)-\frac{1}{b-a}\sum ^b_{s=a+1}\sum ^b_{t=a+1}k(x_s,x_t) \end{aligned}$$

where the $k(\cdot ,\cdot )$ is user-defined kernel function in the form of $R^d*R^d \rightarrow R$ and this formula is inferred by the well-known ”kernel trick”^60^.

The Gaussian kernel is defined by $k(x,y)=exp(-\gamma \Vert x-y\Vert ^2)$ with $x,y\in R^d$and $\gamma >0$.

By using the Gaussian kernel, the Rbf kernel cost function is given by

10 $$\begin{aligned} c_{rbf}(x_{a\ldots b}):=(b-a)-\frac{1}{b-a}\sum ^b_{s=a+1}\sum ^b_{t=a+1}exp(-\gamma \Vert x_s-x_t\Vert ^2) \end{aligned}$$

The detected results of our change point algorithm on all sectors are shown in Supplementary Fig. 5.

### Time series prediction model based on graph learning: TPG

In order to model the recovery process of all sectors and accurately predict the future recovery trend of them, we construct the time series prediction model based on dynamic graph learning, named TPG. Here we provide a more comprehensive introduction of TPG.

### Problem formulation

The training task of TPG is to predict the future daily electricity consumption sequence for each organization with the historical daily electricity consumption sequences. More specifically, X = {$$x_{c,t} $$}$$\in \Re ^{N\times T}$$ stands for the electricity consumption input, where *N* is the number of organizations, and *T* is the number of timestamps. We denote the daily electricity consumption sequence of an organization *c* at timestamp *t* as $$x_{c,t}$$:

11 $$\begin{aligned} x_{c,t} = [e_{c}^i], t*s \le i <t*s + l_{in}, c \in C \end{aligned}$$

Here $l_{in}$ is the length of input sequences (window size), *s* is the slides length, and $e_{c}^i$ is the electricity consumption of organization *c* in the day *i*. Besides, each organization *c* belongs to one type of sector. With $x_{c,t}$, our goal is to predict the organization’ future daily electricity consumption sequence {$y_{c,t}$} at timestamp *t*:

12 $$\begin{aligned} y_{c,t} = [e_{c,t}^i], t*s + l_{in} \le i <t*s + l_{in} + l_{out}, c \in C \end{aligned}$$

where $l_{out}$ is the the length of the future daily electricity consumption sequence that we want to predict.

### Framework

With the electricity consumption data, we first build the dynamic correlation graph to help model the recovery process and accurately capture the future recovery trends. Each node in the learned graph represents an organization in our electricity dataset and each edge represents the influence relationship between the organizations. When building the graph, we have two steps: node feature generation and edge calculation. For the node feature generation, we adopt a Gated Recurrent Unit (GRU) in the temporal learning module to capture the temporal information from organizations’ historical daily electricity consumption, using the final organization embeddings in GRU as node features. For edge calculation, we design a correlation module. We calculate the correlation weights between organizations in the correlation module with an attention mechanism and use the results as the basis to build the graph edges.

Then, on the learned dynamic graph, each organization will aggregate information from its connected organizations based on the learned edge (correlation) weights. In this way, we get the new representation of each organization containing both temporal information and correlation information. Finally, we use a GRU decoder to get the prediction results from these representations.

### Temporal learning module

In this section, we elaborate on the method by which we generate the temporal representation for each organization with daily electricity consumption data. We adopt a GRU as an encoder to model organizations’ temporal information with *X*. For an organization *c*, we generate its temporal representation $p_{c,t}$ at timestamp *t* as follows:

13 $$\begin{aligned} p_{c,t}^s= & {} e_c^s \end{aligned}$$

14 $$\begin{aligned} z_{c,t}^{i}= & {} \sigma (W_z^ih_{c,t}^i + U_z^ih_{c,t}^{i-1}) \end{aligned}$$

15 $$\begin{aligned} r_{c,t}^{i}= & {} \sigma (W_r^ih_{c,t}^i + U_r^ih_{c,t}^{i-1}) \end{aligned}$$

16 $$\begin{aligned} \widetilde{h}_{c,t}^{i}= & {} tanh(W^ih_{c,t}^i + U^i(r_{c,t}^i\odot h_{c,t}^{i-1})) \end{aligned}$$

17 $$\begin{aligned} \hat{h}_{c,t}^{i}= & {} (1 - z_{c,t}^i)\odot h_{c,t}^{i-1} + z_{c,t}^i\odot \widetilde{h}_{c,t}^{i} \end{aligned}$$

18 $$\begin{aligned} p_{c,t}^i= & {} GRU(p_{c,t}^{i-1}, e_c^i), s<i <s + l_{in} \end{aligned}$$

where *z* is the update gate vector to control the information retained in the previous timestamp; $\widetilde{h}$ is the candidate activation vector; the *r* is the reset gate vector to control the generation of candidate activation vector $\widetilde{h}$; finally, we get the output vector *h* by aggregate the candidate activation vector and the previous output vector. In this process, we expand the $e_c^s$ by sliding window sampling time series with window size 7 ($l_{in}$) and slides length 1. *i* denotes the time step in GRU, *s* denotes the first date of the sampled time series.

### Correlation learning module

To capture the organization correlation information to help recovery modeling, we adopt a correlation learning module. The correlation module calculates the correlation weights between organizations, which are the basis to build the graph edges. With the input *X*, we first calculate the dynamic correlation weights *W* of all organizations to another one at each timestamps:

19 $$\begin{aligned} w_{u,v,t}=(Q \cdot x_{u,t}) \odot (K \cdot x_{v,t}) \end{aligned}$$

where $w_{u,v,t}$ is the correlation weight that represents the influence of organization *v* on organization *u* at timestamp *t*, $x_{u,t}$ and $x_{u,t}$ are their corresponding daily electricity consumption sequences. *Q* is a learnable matrix. For each organization, *Q* projects its temporal information of it into a “query” embedding. And *K* is also a learnable matrix that projects the temporal information of other organizations into “key” embeddings. Then we dot product the query embedding and key embeddings to get the correlation weight values which represent how much influence the “key organizations” will have on “query organization”. Because an organization usually only receives influence from a limited number of other organizations, at timestamp *t*, we select the top *k* largest $w_{u,*,t}$ for every organization *u* and drop the other weights. *k* is a hyperparameter in our model. Then we normalized the remained $w_{u,*}$ to get the final correlation weights:

20 $$\begin{aligned} \hat{w_{u,v,t}} = \frac{w_{u,v,t}}{\sum _{v\prime \in N(u)_t}{w_{u,v,t\prime }}} \end{aligned}$$

where $N(u)_t$ denotes the *k* organizations that have the greatest impact on organization *u* at timestamp *t*. With the learnt correlation weights, we build the edges of the graph. For each correlation weight $w_{u,v,t}$, we build a directed edge from node (organization) *w* to node *v* with an edge weight $w_{u,v,t}$, which represents the influence that organization *w* will have on node *v* at time step *t*.

Now we have completed the building of the correlation graph. We denote the built graph as $G_t = (C,E,P_t,W_t)$, where *C* denotes the node set, each node of the directed correlation graph $G_t$ is an organization *c* with its temporal representation $p_{c,t}$. And based on *W*, we build the edge set *E*, each edge is attached with a correlation weight $w_{u,v,t}$.

### Aggregating and predicting module

For each organization (node), to utilize the information of its correlated organizations, we conduct weighted aggregation on the temporal correlation graph $G_t$, and combine the aggregated information with the temporal information of this organization:

21 $$\begin{aligned} r_{c,t} = \sum _{{c\prime } \in N(c)_t}{w_{c,c\prime ,t} \cdot p_{c\prime ,t}} + p_{c,t} \end{aligned}$$

Here we use the edge (correlation) weight which is introduced in the above section as the aggregation weight. After the aggregation, for each organization, we obtain the new representations that simultaneously contain temporal information of itself and that of its correlated organizations whose future sequence may be influenced.

A GRU is then adopted as the decoder to generate the future daily electricity consumption sequences of organizations from the learned representations:

22 $$\begin{aligned} o_c^{s+l_{in} - 1}= & {} r_c \end{aligned}$$

23 $$\begin{aligned} o_c^{i}= & {} GRU(o_c^{i-1},o_c^{i-1}), s + l_{in}<i <s + l_{in} + l_{out} \end{aligned}$$

24 $$\begin{aligned} \hat{e_c^{i}}= & {} MLP(o_c^i) \end{aligned}$$

25 $$\begin{aligned} \hat{y_c}= & {} [\hat{e_c^i}], s + l_{in} \le i <s + l_{in} + l_{out}, c \in C \end{aligned}$$

Following GRU cell, we decode the new compound representation iteratively.

As the prediction of daily electricity consumption is a natural regression task, we use mean square error as our loss function to calibrate our model’s parameters:

26 $$\begin{aligned} loss_c = MSE(y_c, \hat{y_c}) = \frac{1}{2}\cdot (e_c^i - \hat{e_c^{i}})^2, s + l_{in} \le i <s + l_{in} + l_{out}, c \in C \end{aligned}$$

The MSE loss measures the distance between the real daily electricity consumption and the estimated consumption.

### Experimental data

Our experimental data is generated based on the electricity consumption data from January to August 2019 and January to August 2020 (487 days in total). In our experiment, we sample the data with a sliding window. We set the slides length *s* as 7, the length of input sequences $l_{in}$ as 56 and the length of output sequences $l_{out}$ as 14. As a result, we get 48 time series samples for every organization. We split the data into 40 for training and 8 for testing sets. We use the first 20 sampled sequences of each year as the training set and the last four as the testing set to ensure that there is no label leakage.

### Training

The optimization of the parameters was performed by the Adam algorithm with a learning rate of 1e-3 and weight decay of 1e-4. The TPG tasks are conducted for 500 epochs, and we selected the model with the lowest loss on the testing set. The selected correlation number *k* is set as 10. The hidden size is 64. Lastly, we build our model using *PyTorch 1.6* and train it on an *NVIDIA GeForce RTX 2080 Ti* GPU.

### Analysis with TPG

#### Experiment results and the baseline

As shown in Fig. 4b, our trained model can fit the future daily electricity consumption well. This result shows our TPG model could model the recovery process and perceive the sector future recovery trend through the past electricity consumption information (Supplementary Table 3). We also examine the effectiveness of our correlation learning module by comparing our experiment results with a single GRU sequence to sequence model (remove the correlation learning part). As shown in Fig. 5, our model obviously outperforms the GRU seq2seq model (reduces 16% on MSE loss on average after 500 epochs), which illustrates our correlation module does capture the organization correlation information that is helpful to the recovery modeling.

Experiments are repeated at least five times, and we report the mean result.

#### Simulation experiments

We conduct simulation experiments with our trained model on the daily electricity consumption data ranging from Jan. 08, 2020 to Mar. 24, 2020, which contains both the lockdown period and recovery period. We fixed all parameters of trained TPG in the simulation experiments. For each sector, we assume that the government has given greater support to this sector when the economy started to recover after the lifting of the COVID-19 lockdown (05 Feb. 2020). As shown in Fig. 4b, we construct simulation electricity consumption series of each sector by doubling the speed of its recovery from 05 Feb. 2020. More specifically, in each of our experiments, we change the time series inputs of all organizations belonging to a particular sector to our simulation values:

27 $$\begin{aligned} E_c^{week j} = E_c^{week (j-5)*2+j}, j \in [5,9) \end{aligned}$$

And we leave the time series of organizations in other sectors unchanged. Then we input the simulation data into the model to get the predicted future recovery trend, so as to see how the change of policy support for a certain sector will affect all the sectors of the society (shown in Fig. 4 and Supplementary Fig. 6).

## Supplementary Information

Supplementary Information.

## Data Availability

The data that support the findings of this study are available from the State Grid Corporation of China, but restrictions apply to the availability of these data, which were used under license for the current study, and so are not publicly available. Data are however available from the authors upon reasonable request and with permission of the State Grid Corporation of China.
