# Supplementary material for: Unfolding and modeling the recovery process after COVID lockdowns
Source: Sci Rep. 2023 Mar 13;13:4131. doi: 10.1038/s41598-023-30100-5 (PMC10009856; doi:10.1038/s41598-023-30100-5)
Supplement: Supplementary file 1 — Supplementary Information. [file 41598_2023_30100_MOESM1_ESM.pdf]

Unfolding and Modeling the Recovery Process after COVID Lockdowns

Xuan Yang,[1] Yang Yang,[1*] Chenhao Tan,[2] Yinghe Lin,[3]
Zhengzhe Fu,[1] Fei Wu,[1] Yueting Zhuang[1]

[1]Zhejiang University
[2]University of Chicago
[2]Zhejiang Huayun Info-Tech Co., Ltd.

Supplementary materials

Sector type	Original category	Sector type	Original category	Sector type	Original category
Manufacturing	-Manufacturing of special instruments<br>-Shoe sector<br>-Chinese patent medicine production<br>-Processing of Chinese herbal pieces<br>-Photovoltaic equipment and components manufacturing<br>-Other unspecified manufacturing<br>-Manufacture of technical textile products<br>-Dairy product manufacturing<br>-Manufacture of other instruments<br>-Other agricultural and sideline food processing<br>-Amusement equipment and entertainment product manufacturing<br>-Other unspecified general equipment manufacturing<br>-Manufacturing of other rubber products<br>-Manufacturing of other glass products	Manufacturing	-Communication equipment manufacture<br>-Tire Manufacture<br>-General instrumentation manufacture<br>-General parts manufacture<br>-Mining, metallurgy, construction special equipment manufacture<br>-Wire rope and its products manufacture<br>-Metal daily necessities manufacture<br>-Metal processing machinery manufacture<br>-Metal furniture manufacture<br>-Metal scrap and scrap processing<br>-Metal surface treatment and heat treatment processing<br>-Knitted or crocheted fabric and its products manufacturing<br>-Railway transportation equipment manufacturing<br>-Foundry and other metal products manufacturing	Infrastructure	-Gas production and supply sector<br>-Public lighting<br>-Other water conservancy management<br>-Urban park management<br>-Other water treatment, utilization and distribution<br>-Solar power generation<br>-Municipal facilities management<br>-Water resources management<br>-Sewage treatment and recycling<br>-Tourist scenic spot management<br>-Environmental sanitation management<br>-Environmental management<br>-Ecological protection<br>-Greening management

Sector type	Original category	Sector type	Original category	Sector type	Original category
	-Manufacturing of transportation equipment and other transportation equipment		-Boiler and auxiliary equipment, other prime mover equipment manufacturing		-Tap water production and supply
	-Other electronic equipment manufacturing		-Non-metallic waste and scrap processing		-Flood control facility management
	-Other electrical machinery and equipment manufacturing		-Non-professional audio-visual Equipment manufacturing	Construction	-Land management sector
	-Other transmission and distribution and control equipment manufacturing		-Containers and metal packaging container manufacturing		-Other real estate sector
	-Processing of timber and timber components for construction		-Wind energy prime mover equipment manufacturing		-Building demolition and site preparation activities
	-Other food manufacturing		-Beverage manufacturing		-Real estate agency services
	-Manufacturing of veterinary drugs	Real estate	-Housing construction sector		-Real estate development and operation
	-Gasoline and diesel vehicle manufacturing		-Civil engineering construction sector		-Real estate leasing operation
	-Manufacture of electronic components and special electronic materials		-Construction and installation sector	Retail	-Specialized retail of hardware, furniture and interior decoration materials
	-Cutting tool manufacturing		-Other unspecified construction sector		-Other wholesale sectors
	-Manufacture of gypsum, cement products and similar products		-Architectural decoration and decoration sector		-Other automobiles, motorcycles, spare parts and fuel and other power sales
	-Manufacturing of chemical preparations	Catering	-Others catering sector		-Stalls, non-stores and other retail trade
	-Manufacture of special equipment for electronic and electrical machinery		-Fast food service		-Specialized retail of medicine and medical equipment
	-Medical equipment and equipment manufacturing		-Dinner service		-Specialized retail of household appliances and electronic products
	-Manufacture of hygiene materials and medical supplies	Scientific and technical service	-Agricultural scientific research and experimental development		-Specialized retail of cultural, sporting goods and equipment
	-Manufacturing of paints, inks, pigments and similar products Services and other specialized facilities		-Industrial and professional design and other professional technical services		-Specialized retail of machinery and equipment, hardware products and electronic products
	-Cigarette manufacturing		-Geological exploration		-Integrated retail
	-Synthetic fiber manufacturing		-Medical research and experimental development		-Wholesale of textiles, clothing and household goods
	-Manufacture of electric wires, cables, optical cables and electrical equipment		-Engineering and technical research and experimental development		-Specialized retail of textiles, clothing and daily necessities
	-Plastic packaging box and container manufacturing		-Engineering technology and design services		-Mineral products, building materials and chemicals wholesale of products

Sector type	Original category	Sector type	Original category	Sector type	Original category
	-Environmental protection, postal services, social public services and other special equipment		-Technical extension services		-Wholesale of agricultural, forestry, animal husbandry and fishery products
	-Manufacture of metal products for construction and safety		-Natural scientific research and experimental development		-Specialist retail sale of food, beverages and tobacco products
	-Manufacturing of plastic parts and other plastic products		-Quality inspection technical services		-Wholesale of food, beverages and tobacco products
	-Manufacture of building materials such as bricks and stones	Resident service	-Motor vehicles, electronic products and daily product repair sector	Transportation	-Air transport sector
	-Manufacture of home textile products		-Other residential services sector		-Road passenger transport
	-Industrial robot manufacturing		-Other unspecified service sector		-Other storage sector
	-Manufacture of arts and crafts and etiquette articles		-Funeral services		-Other logistics services
	-Plastic film manufacturing		-Barber and beauty services		-Other water transport auxiliary activities
	-Manufacturing of other metal tools	Education	-Secondary education		-Urban public transport
	-Culture and office machinery manufacturing		-Elementary education		-Water passenger transport
	-Cultural and educational office supplies manufacturing		-Skill training, educational assistance and other education		-Water freight transport
	-Convenience food manufacturing		-Pre-school education		-Electrified railway
	-Daily use chemical product manufacturing		-Higher education		-Loading, unloading and handling
	-Daily use and medical rubber product manufacturing	Health	-Professional public health services		-Warehousing of grain, cotton and other agricultural products
	-Daily plastic manufacturing		-Other health activities		-Transportation agency
	-Manufacture of daily miscellaneous goods		-Hospitals		-Road freight transportation
	-Non-ferrous metal calendaring		-Primary medical and health services		-Road transportation auxiliary activities
	-Apparel manufacturing	Entertainment	-Sports organizations		-Postal basic services
	-Wooden furniture manufacturing		-Sports venue facilities management		-Railway passenger transportation
	-Woven clothing manufacturing		-Fitness and leisure activities		-Railway transportation auxiliary activities
	-Cotton textile and printing and dyeing finishing		-Other cultures	Accommodation	-General hotels
	-Rubber parts manufacturing		-Publishing		-Other accommodations
	-Pesticide manufacturing		-Museums		-Tourist hotels
	-Auto parts and accessories manufacturing		-Printing	Information Service	-Internet information service
	-Foam plastic manufacturing		-Libraries and archives		-Internet data services
	-Manufacturing of pumps, valves, compressors and similar machinery		-Cultural relics and intangible cultural heritage protection		-Information processing and storage support services

Sector type	Original category	Sector type	Original category	Sector type	Original category
	-Manufacturing of sanitary ceramic products		-Radio and television transmission services		-Information system integration services
	-Other electricity production		-Film and television program production		-Other information technology services
	-Baked goods manufacturing		-Radio		-Telecommunications
	-Manufacturing of plastic sheets, tubes and profiles		-Literary and artistic creation and performance		-Software development
	-Material handling equipment manufacturing		-Journalism	Financial	-Personal insurance
	-Manufacture of knives, scissors and similar household metal tools		-Martyrs cemeteries and memorial halls		-Insurance asset management
	-Lighting equipment manufacturing		-Television		-Other unlisted financial activities
	-Bio-based material manufacturing		-Mass cultural and sports activities		-Holding company services
	-Bio-pharmaceutical product manufacturing		-Art performance venues		-Securities market services
	-Toy manufacturing	Management	-Social security		-Property insurance
	-Manufacturing of chemical raw materials		-Chinese communist party organs		-Currency banking services
	-Manufacture of electronic devices		-International organizations		-Financial trust and management services
	-Manufacture of electrical machines		-Social work without accommodation		-Non-monetary banking services
	-Basic chemical raw material manufacturing		-People’s political consultative conference	Business Service	-Conference, exhibition and related services
	-Specialty chemical product manufacturing		-People’s courts and people’s procuratorates		-Other capital market services
	-Manufacturing of household electrical appliances		-Other state institutions		-Other technology promotion services
	-Refined tea processing		-State power agencies		-Other business services
	-Cellulose fiber raw material and fiber manufacturing		-State administrative agencies		-Consultation and investigation
	-Paper product manufacturing		-Religious organizations		-Security protection services
	-Structural metal product manufacturing		-Social work that provides accommodation		-Provision of construction equipment services
	-Feather (down) processing and product manufacturing		-Villagers self-governance Organize		-Machinery and equipment operating leasing
	-Grain grinding		-social groups		-Legal services
	-Bicycle and disabled seat vehicle manufacturing		-Community resident self-governing organizations		-Property management
	-Computer manufacturing		-Organization management services		-General management services
	-Binding and other printing services activities		-Mass groups		

Supplementary Table 1: The sector classification table. The original sector category is offered by the State Grid Corporation of China, which is the third level sector category in ’Industrial classification for national economic activities’.

Issued date	department	contents
23 Jan. 2020	Zhejiang Provincial Government	Zhejiang provincial government decided to launch the first-level response to major public health emergencies.
24 Jan. 2020	Zhejiang Provincial Department of Culture and Tourism	Hangzhou canceled and suspended folk, religious and cultural festivals and other activities: canceled the Buddhist cultural tourism activities in the Spring Festival; canceled students' collective holiday activities; closed the scenic spots and museums of West Lake; suspended group dinners in rural family banquet centers.
27 Jan. 2020	Hangzhou Municipal Government	Delayed the resumption of business and the start of school.
30 Jan. 2020	Leading group for prevention and control of COVID-19	Temporarily closed some highway exits.
1 Feb. 2020	Leading group for prevention and control of COVID-19	Stopped some bus routes and extended the interval time of urban subways.
2/3 Feb. 2020	Hangzhou Municipal Government	Hangzhou and its districts successively issued "the most forbidden orders": "Ten Uniforms", "Three No Four Strict and Five Must Check" and "Ten Strict Controls" and other announcements.
4 Feb. 2020	Hangzhou Municipal Government	On February 4, Hangzhou took the lead in tackling the "one person, one code, scanning code to determine" plan. On February 11, the health code was officially launched, and the construction of the "health code" management system was accelerated.
5 Feb. 2020	Zhejiang Provincial Department of Transportation	Clarified the classification and classification to ensure the transportation needs of emergency materials of key enterprises, and ensure the priority protection and free passage of relevant vehicles.
Feb-Apr. 2020	Zhejiang Provincial Department of Economy and Information Technology	To support small and micro enterprises to tide over difficulties, 17 policies had been issued, which can be summed up as "four increased supports": increased support for small and micro enterprises to reduce factor costs, finance and taxation, finance, and foreign trade exports.
10 Feb. 2020	Zhejiang Provincial Government	The resumption of work in the city's enterprises implemented the return to work declaration and filing system, and the resumption of work and production was promoted in an orderly manner according to the location, time, and classification.
11 Feb. 2020	Hangzhou Municipal Government	Hangzhou introduced the "1+12" policy to help enterprises resume work and production. "1" refers to the implementation of relevant policies at the central and provincial levels, and "12" refers to 12 policies that benefit enterprises: reduced corporate financing costs; waived corporate guarantee fees; temporarily reduced Medical insurance rates; reduced the ratio of corporate housing provident fund deposits; reduced or exempt corporate rent; subsidized commercial and trade service companies; increased support for property companies; issued rental subsidies for corporate employees; coordinated the solution of transitional accommodation for returning workers; guided the solution of "nursing difficulties" for dual-earner families; fully guaranteed the procurement and supply of anti-epidemic materials; increased legal aid efforts.
20 Feb. 2020	Zhejiang provincial government	Except for the entertainment sector and some service industries, most companies had resumed work.
13 Mar. 2020	Hangzhou Municipal Bureau of Culture, Radio, Television and Tourism, Hangzhou Municipal Bureau of Finance	To promote the revival of the tourism market, Hangzhou had introduced eight measures: setted up rescue funds for the tourism sector; encouraged hotels to offer discounts on price; encouraged A-level scenic spots to implement free opening; encouraged travel agencies to increase the introduction of tourists; supported "contactless" tourism services Scenario application; encouraged "Hangzhou people to visit Hangzhou"; speeded up the temporary refund of tourism service quality guarantee; increased legal assistance to travel agencies.
18-31 Mar. 2020	Leading group for prevention and control of COVID-19	The Hangzhou Municipal Government tried to implement free bus and subway time-period measures, and continued to implement bus and subway prevention and control measures.
23 Mar. 2020	Hangzhou Education Bureau	Off-campus training institutions were promoting the resumption of work and classes in an orderly manner.
27 Mar. 2020	Hangzhou Municipal Government	Hangzhou had begun to issue consumer coupons totaling 1.68 billion yuan to all employees in Hangzhou.

Issued date	department	contents
3 Apr. 2020	Leading group for prevention and control of COVID-19	Schools of all levels and types would open in order from grade to grade within three weeks from April 13, 2020.
14 Apr. 2020	Hangzhou Municipal Party Committee Propaganda Department, Hangzhou Municipal Finance Bureau	Subsidized the public welfare performances of literary and art troupes; reduced the financing cost of cultural enterprises; subsidized the participation fees of cultural enterprises; helped cultural enterprises to solve difficulties.
4 May. 2020	Leading group for prevention and control of COVID-19	Colleges and universities across the province would start school from April 26 to May 10.
9 May. 2020	Leading group for prevention and control of COVID-19	After verification, all kinds of off-campus training institutions in Hangzhou could resume off-line training since May 9th, if they meet the standards of resumption of classes (retraining).
18 May. 2020	Leading group for prevention and control of COVID-19	All kinds of kindergartens across the city had started school one after another.
22 May. 2020	Leading group for prevention and control of COVID-19	Entertainment and leisure places such as theaters could be opened in an orderly manner by making reservations and limiting current.
2 Jun. 2020	Leading group for prevention and control of COVID-19	Gradually and orderly reopened places for religious activities.
16 Jun. 2020	Leading group for prevention and control of COVID-19	Strengthened the control of food circulation and investigate potential food safety hazards. Increased the daily supervision and inspection of catering units.
14 Jul. 2020	Ministry of Culture and Tourism	Except for medium and high-risk areas, inter-provincial (regional, municipal) group travel would be resumed.
16 Jul. 2020	National Film Bureau	Promoted the reopening of cinemas in an orderly manner.

Supplementary Table 2: We have collected the policies that mainly affect these 17 sectors in Hangzhou.

Epoch	100	200	300	400	500
GRU	3.4307141	3.777505	3.7792249	3.645605	3.3568
GRU	3.4576638	3.3798978	3.4166617	3.370823	3.3515115
GRU	3.4902925	3.3480632	3.4429383	3.5454762	3.357866
GRU	3.4078832	3.5348659	3.423835	3.441637	3.4517586
GRU	3.3835907	3.3667076	3.459504	3.3569682	3.4272494
TPG	3.1193743	3.13383	3.177925	3.2520874	3.3169534
TPG	2.9864151	3.0294094	3.093755	2.8886776	3.034157
TPG	2.8584437	3.084448	2.9553668	3.2733083	2.8418617
TPG	2.9539533	2.9836297	2.8260555	3.068309	2.826255
TPG	3.079696	3.0674415	2.8529513	2.8119133	2.8507543

Supplementary Table 3: Experiment results of TPG on electricity consumption dataset. The values in the table represent the MSE loss results. The TPG can accurately predict the future recovery trend and get much better performance than GRU.

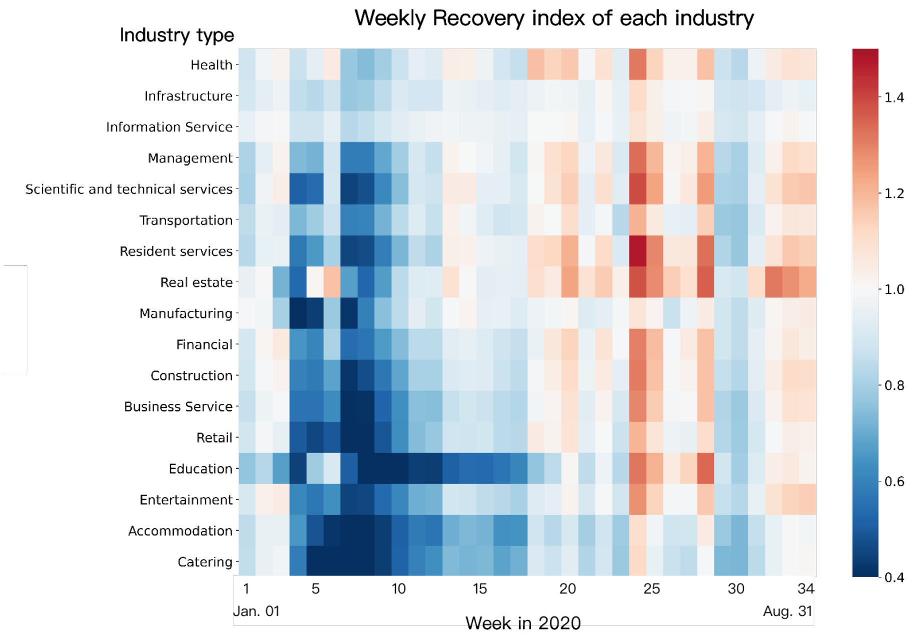

Supplementary Figure 1: January-July 2020 Unadjusted recovery index: Year-on-year (2020-on-2019) change in weekly electricity consumption.

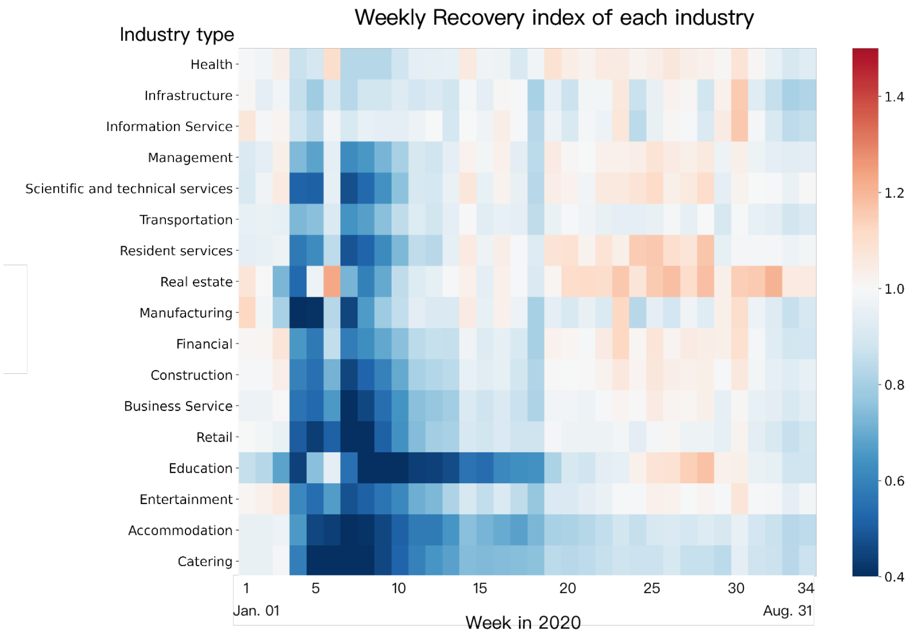

Supplementary Figure 2: January-July 2020 recovery index, adjusted with temperature factor.

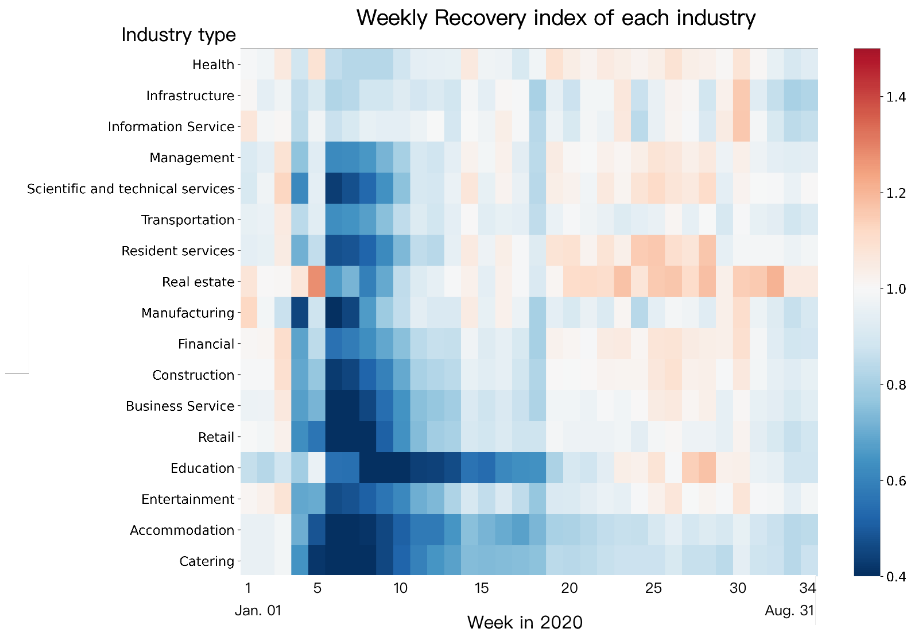

Supplementary Figure 3: January-July 2020 recovery index, adjusted with temperature, lunar festival factors.

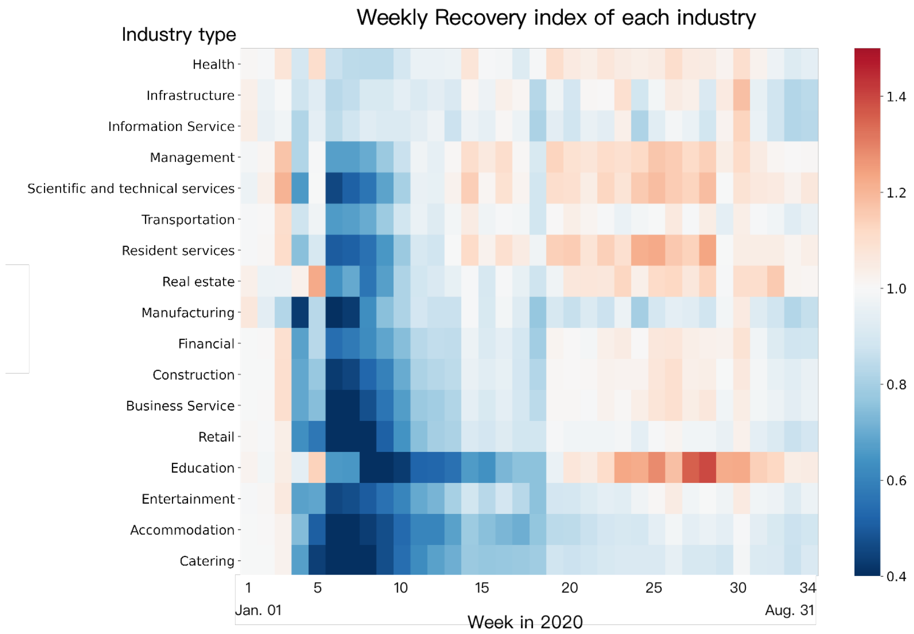

Supplementary Figure 4: January-July 2020 recovery index, adjusted with temperature, lunar festival and development level factors.

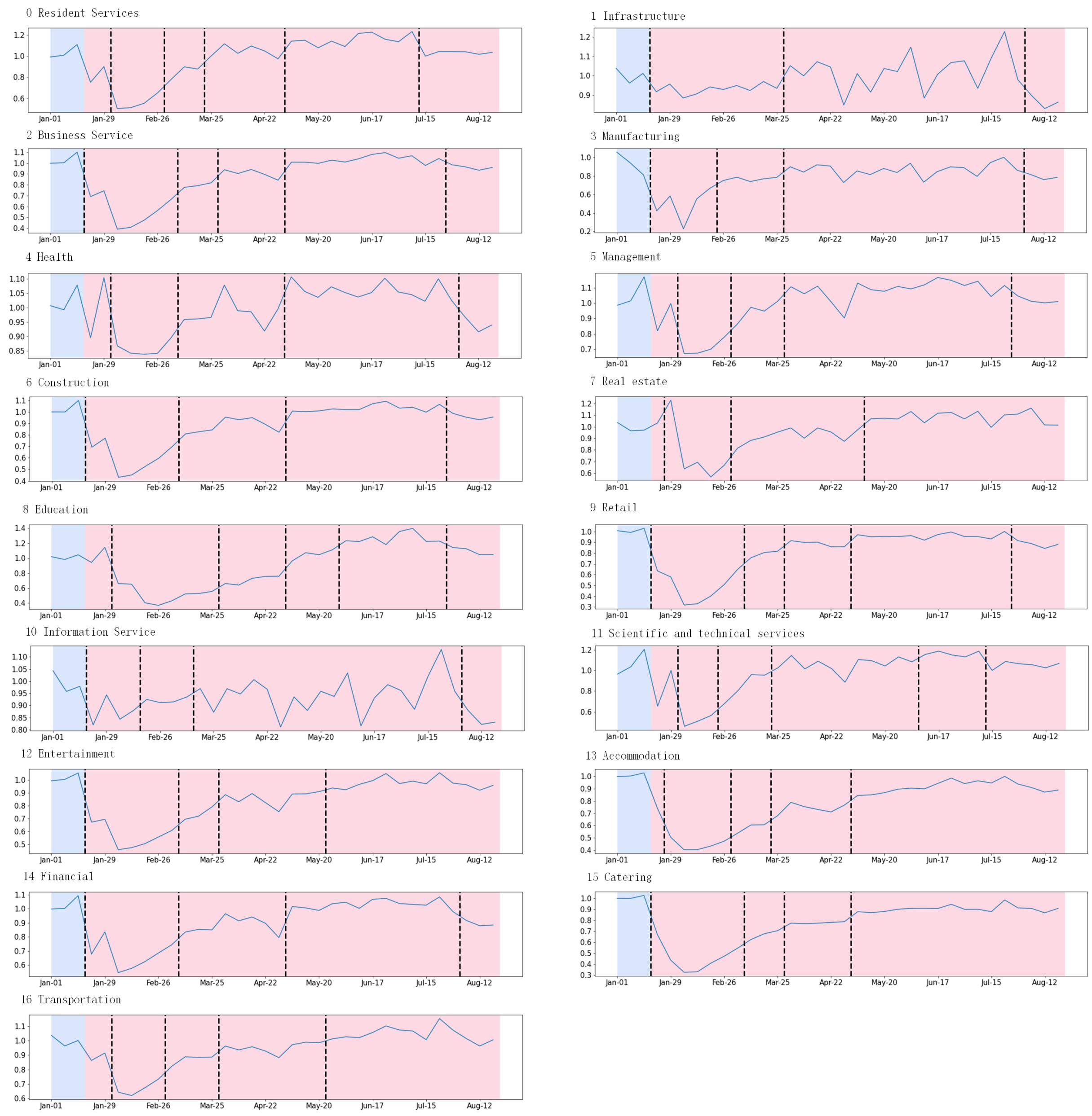

Supplementary Figure 5: The detected results of change point algorithm on the weekly recovery index of all sectors from Jan. 01, 2020 to Aug. 31, 2020. The blue areas represent dates prior to the outbreak (lockdown). The pink area represents the date after the outbreak (lockdown).

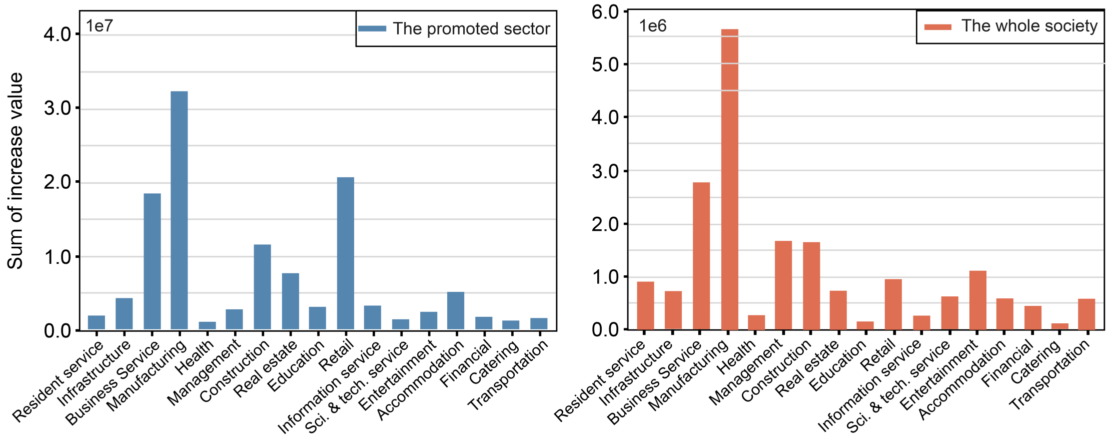

Supplementary Figure 6: The impact on the future recovery if we provide supports to different sectors in the simulation experiments. The left figure is the sum of increase value of electricity consumption for the next 14 days on a sector that caused by the added policy support on itself. The right figure is the sum of increase value of electricity consumption for the next 14 days on all sector except the policy-promoted sectors.
